# Evaluation of hypoglycemic and anti-hyperglycemic potential of *Tridax procumbens *(Linn.)

**DOI:** 10.1186/1472-6882-9-48

**Published:** 2009-11-29

**Authors:** Hemant Pareek, Sameer Sharma, Balvant S Khajja, Kusum Jain, GC Jain

**Affiliations:** 1 Department of Zoology, S K Govt (PG) College, Sikar (Rajasthan), India; 2 Department of Zoology, Govt (PG) College Sawai Madhopur (Rajasthan), India; 3 Center For Advanced Studies, Department of Zoology, University of Rajasthan, JLN Marg, Jaipur-302 004, India

## Abstract

**Background:**

Diabetes is a metabolic disorder affecting carbohydrate, fat and protein metabolism. *Tridax procumbens *Linn. (Family-Asteraceae; common name-Dhaman grass) is common herb found in India. Traditionally, the tribal inhabitants of Udaipur district in Rajasthan (India) uses the leaf powder (along with other herb) orally to treat diabetes. There is a need to evaluate extracts of this plant in order to provide scientific proof for it's application in traditional medicine system.

**Methods:**

Extraction of whole plant of *T. procumbens *using 50%methanol. The extract was tested for acute and sub-chronic anti-hyperglycemic activity in alloxan induced diabetic rats and for acute toxicity test among normal rats. Observations on body weight as well as on the oral glucose tolerance levels were also recorded.

**Results:**

Oral administration of acute and sub chronic doses (250 and 500 mg/kg b.wt.) of *T. procumbens *extract showed a significant (p < 0.05) reduction in fasting blood glucose levels in diabetic rats, however the decline in blood sugar levels in normal rats was not observed. In acute study the maximum percent blood glucose reduction (68.26% at 250 mg/kg and 71.03% at 500 mg/kg body weight) in diabetic rats was observed at 6 h. The anti-hyperglycemic effects were not dependent of dose and the OGTT and Body weight supported the antihyperglycemic action of the drug. The results of anti-diabetic effect of *T. procumbens *were compared with the reference standard drug Glibenclamide (10 mg/kg b.wt.).

**Conclusion:**

These test results support traditional medicinal use of, *T. procumbens *for the treatment of diabetes mellitus with corrections in body weight and oral glucose tolerance and no visible signs or symptoms of toxicity in normal rats indicating a high margin of safety. These results warrant follow-up through bioassay-directed isolation of the active principles.

## Background

Diabetes is a metabolic disorder affecting carbohydrate, fat and protein metabolism. It is affecting nearly 10% of the population world wide[1]. The frequency of the diabetes will escalate worldwide, with a major impact on the population of developing countries[2]. The Current studies in India indicate that there is an alarming rise in prevalence of diabetes which has gone beyond epidemic form to a pandemic one[3]. Currently India has got the largest number of diabetics and is being called as diabetic capital of the world[4].

The Diabetes Control and Complications Trial (DCCT) demonstrated that tight control of blood glucose is effective in reducing clinical complications significantly, but even optimal control of blood glucose could not prevent complications suggesting that alternative treatment strategies are needed[5]. The available therapies for diabetes include insulin and many oral hypoglycemic agents, such as biguanids and sulfonylureas[6]. Treatment with sulfonylureas and biguanids is also associated with side effects and fail to significantly alter the course of diabetic complications[7]. The search for new pharmacologically active agents obtained by screening natural sources such as medicinal plants or their extracts has led to the discovery of many clinically useful drugs that play a major role in the treatment of human diseases. A number of medicinal plants and their formulations are used for treating diabetes in folklore/Ayurvedic medicine system as well as in ethnomedicinal practices[8]. WHO (1980) has also recommended the evaluation of the plants effective and in conditions where we lack safe modern drugs[9]. This leads to increasing demand for herbal products with anti-diabetic activity and less side effects.

*Tridax procumbens *Linn. (Family-Asteraceae; common name-Dhaman grass) is common herb found in India. The whole plant and seeds are reported to be used to treat various aliments, such as bronchial catarrh, dysentery, diarrhea, preventing hair loss, and to check hemorrhage from cuts [10,11]. Pharmacological studies have shown that *T. procumbens *possess properties like-anti inflammatory, hepatoprotective, wound healing, Immunomodulatory, antimicrobial, antiseptic, and hypotensive, bradycardiac effects [12-16]. Earlier workers [17-20] have already reported the presence of *dexamethasone*, *luteoline*, *glucotureolin*, *β-sitosterol*, *flavone, glycoside *and *quercetin *in this plant. Very little information is available regarding hypoglycemic/anti-hyperglycemic property of Tridax.

Traditionally, the tribal inhabitants of Udaipur district in Rajasthan (India) uses the leaf powder (along with other herb) orally to treat diabetes [21]. Until now no scientific investigation had been carried out to shed light on the anti-diabetic, hypoglycemic property of *Tridax procumbens*. Thus, in order to validate the tribal use of the plant as an anti-diabetic, the objective of the present study was to study the effects of the methanolic (50%) extract from whole plant of *Tridax procumbens *on body weight, Fasting blood glucose as well as on the oral glucose tolerance levels, in the models of normal and alloxan-induced diabetes, in rats.

## Methods

### Plant Material

*Tridax procumbens*, whole plant was collected from the Udaipur district during the month of September-October, and was identified and authenticated from the department of Botany, University of Rajasthan, Jaipur. A voucher specimen (RUBL No. 20534) was retained in the Herbarium of the department of Botany, University of Rajasthan, Jaipur.

### Preparation of Extract

The shed-dried plant material was coarsely powdered (6.9 kg) and extracted with 50% methanol using soxhlet apparatus for 36 hrs. The resulting mixture was filtered and the filtrate was evaporated in an oven at 40°C to get the dry residue (7.86 g). The resultant residue was used as 'drug' in experiments.

### Chemicals

Alloxan monohydrate was obtained from Sigma Chemical Co. (St. Louis, MO., USA). Glibenclamide tablets (Daonil; Aventis Pharma. Ltd., India) were procured from the authorized distributor of the company. All other chemicals used were of analytical grade.

### Experimental Animals

Adult healthy male albino rats of Wistar strain weighing 140-160 g, obtained from IVARI Izatnagar, Bareli (U.P.) were used after acclimatization for 14 days for this study. The animals were housed in polypropylene cages under standard husbandry conditions (12 hrs light/dark cycle: 25 ± 3°C). Rats were provided water and pellet diet (Hindustan Lever Ltd., Bangalore, India.) *ad libitum*. The study was conducted after the approval from the institutional ethical committee for animal care.

### Induction of Experimental Diabetes in Rats

After fasting, diabetes was induced by a single *intraperitoneal injection *of 120 mg/kg body weight of 'Alloxan monohydrate' in distilled water. The animals were allowed to drink 5% glucose solution overnight to overcome the drug-induced hypoglycemia. These animals were tested for diabetes after 15 days and animals with blood glucose (fasting) range 300 - 450 mg/dl were selected for experimentation.

### Experimental Protocol

Animals were divided into seven groups of 6 rats each.

**Group I**: Rats served as normal-control and received the vehicle (0.5 ml distilled water/day/rat)

**Group II**: Rats (normal) were administered *T. procumbens *(250 mg/kg b.wt./day) in distilled water as a fine aqueous suspension orally.

**Group III**: Rats (normal) were administered *T. procumbens *(500 mg/kg b.wt./day) in distilled water as a fine aqueous suspension orally.

**Group IV**: Rats served as diabetic-control and received the vehicle (0.5 ml distilled water/day/rat)

**Group V**: Rats (diabetic) were administered *T. procumbens *(250 mg/kg b.wt./day) in distilled water as a fine aqueous suspension orally.

**Group VI**: Rats (diabetic) were administered *T. procumbens *(500 mg/kg b.wt./day) in distilled water as a fine aqueous suspension orally.

**Group VII**: Rats (diabetic) were administered *Glibenclamide *(10 mg/kg b.wt./day) in distilled water as a fine aqueous suspension orally.

All the rats were fasted for 16 hr. before experimentation, but allowed free access to water.

### Acute Dose Study

All the rats received single dose treatment in all groups.

**1. Blood Sugar Estimation**: Blood samples were collected by tail vein puncture just prior to drug administration and at 1/2, 1, 2, 4, 6 and 8 hrs. The blood glucose was estimated by 'One touch-ULTRA' glucometer (Johnson & Johnson company, USA).

**2. Acute Toxicity Evaluation in Rats**: The methanolic extract was tested for its acute and short-term toxicity (if any) in normal rats. To determine acute toxicity of a single oral administration of herbal drug, different doses of the drug (0.25--5.0 g/kg) were administrated to different groups of rats (8 rats in each group with 4 male and 4 female). Mortality and general behavior of the animals were observed periodically for next 48 h. The animals were observed continuously for the initial 4 h and intermittently for the next 6 h and then again for 24 h and 48 h after the drug administration. The parameters observed were grooming, mood, hyperactivity, sedation, loss of righting reflex, respiratory rate and convolutions.

### Sub-chronic Dose study

All the rats received treatment for 30 days in all groups.

**1. Body weight**: Body weight was measured at the time of alloxan-dosing. After 15 d of alloxan-dosing, the body weight of all the rats was measured once a week with at sacrifice (30 d). Before blood collection and at sacrifice day experimental animals were overnight fasted (water was not restricted) to reduce the erratum of feeding.

**2. Fasting Blood Glucose (FBG) Estimation**: Fasting Blood Glucose was measured at the time of alloxan-dosing. After 15 d of alloxan-dosing, the Fasting Blood Glucose of all the rats was measured once a week with at sacrifice (30 d). Blood samples were collected by tail vein puncture just prior to drug administration and at 7, 15, 21 and 30 days. The blood glucose was estimated by 'One touch-ULTRA' glucometer (Johnson & Johnson company, USA). The results were expressed in terms of mg/dl of blood.

**3. Oral Glucose Tolerance Test (OGTT)**: Prior to an OGTT all the rats were fasted for 16 h Distilled water (control), a reference drug glibenclamide (10 mg/kg b.wt.) or each of the two different doses of Tridax procumbens extract (250 and 500 mg/kg b.wt.) were then orally administered to respective groups of 6 rats each. 30 min. later, glucose (3 g/kg) was orally administered to each rat with a feeding syringe. Blood samples were collected from the tail vein by tail milking at -30(just before the extract and glibenclamide administration), 0 (just before the oral administration of glucose), 30, 60, 90 and 120 min. after glucose load.

### Statistical Analysis

Results were expressed as mean ± SEM. Data were analyzed with one way ANOVA for the comparison between groups, followed by Tukey as a *post hoc test*. The significance level was set at p < 0.05.

## Results

### Acute dose study

1. Effect on Normal Rats

The results of effect oral administration of the plant extract on normal rats are shown in Table 1. The effect of both the doses of 50% methanolic extract of *T. procumbens *on fasting blood glucose levels in normal rats were assessed at different time intervals. 250 mg/kg b.wt. and 500 mg/kg b.wt. doses of *T. procumbens *did not cause any significant change in blood sugar levels.

**Table 1:** Variation in blood glucose levels after oral administration of T. *procumbens*in normal rats

	Normal Control	Normal+ Extract (250 mg/kg)	Normal+ Extract (500 mg/kg)	Normal+ Glibenclamide (10 mg/kg)
0 h	87.16 ± 0.93^a^	84.33 ± 0.67^a^	84.66 ± 0.58^a^	90.16 ± 1.18^a^
1/2 h	85.66 ± 1.10^a^	81.83 ± 1.97^a^	90.66 ± 1.12^a^	87.33 ± 1.90^a^
1 h	88.33 ± 1.90^a^	87.50 ± 1.18^a^	89.00 ± 1.05^a^	84.00 ± 1.10^a^
2 h	88.16 ± 0.66^a^	85.50 ± 2.61^a^	83.33 ± 2.61^a^	81.66 ± 0.66^b^
4 h	84.00 ± 0.61^a^	79.66 ± 1.39^a^	85.16 ± 0.66^a^	73.50 ± 1.10^b^
6 h	85.83 ± 1.05^a^	81.33 ± 0.61^a^	82.16 ± 0.93^a^	62.10 ± 3.60^b^
8 h	84.00 ± 1.10^a^	82.50 ± 1.57^a^	80.83 ± 1.05^a^	56.18 ± 4.50^b^

Values represent mean ± SEM (n = 6).

*Values for a given group in a row followed by same letter as superscript are not significantly different according to Tukey's multiple comparison procedure (at P < 0.05).

2. Effects on Alloxan Induced Diabetic Rats

The results are presented in Table 2 showed that; in the diabetic rats, the fasting blood glucose levels were 4-5 times higher than that of the normal rats. A sharp decline in blood sugar level was observed from 2^nd ^hour after the treatment in both 250 and 500 mg/kg doses of *T. procumbens *and also in glibenclamide (10 mg/kg) treated rats. The percentage blood glucose reduction with 250 and 500 mg/kg doses of *T. procumbens *and glibenclamide (10 mg/kg) was observed maximum at 6 h was 68.26,71.03 and 57.29% respectively. Both the doses of the extract treatment (250 and 500 mg/kg b.wt.) showed more effective lowering of blood sugar then the standard drug viz. glibenclamide (10 mg/kg).

**Table 2:** Variation in blood glucose levels after oral administration of T. *procumbens*in alloxan-induced diabetic rats

	Normal Control	Diabetic Control	Diabetic+ Extract (250 mg/kg)	Diabetic+ Extract (500 mg/kg)	Diabetic+ Glibenclamide (10 mg/kg)
0 h	87.16 ± 0.93^a^	446.60 ± 3.30^b^	406.80 ± 5.18^b^	395.60 ± 3.27^b^	369.70 ± 4.32^b^
1/2 h	85.66 ± 1.10^a^	440.10 ± 4.15^b^	394.60 ± 6.12^c^	389.12 ± 7.90^c^	370.50 ± 3.10^c^
1 h	88.33 ± 1.90^a^	453.60 ± 4.52^b^	336.80 ± 12.90^c^ (17.20)	300.50 ± 10.47^c^ (24.03)	321.10 ± 4.80^c^ (13.14)
2 h	88.16 ± 0.66^a^	436.60 ± 3.90^b^	313.10 ± 15.04^c^ (23.03)	289.70 ± 8.05^d^ (26.76)	264.80 ± 12.66^d^ (28.37)
4 h	84.00 ± 0.61^a^	425.30 ± 6.72^b^	219.70 ± 13.13^c^ (45.99)	188.00 ± 16.22^d^ (52.47)	218.10 ± 16.21^d^ (41.00)
6 h	85.83 ± 1.05^a^	439.30 ± 3.35^b^	129.10 ± 11.05^c^ (68.26)	114.60 ± 9.51^c^ (71.03)	157.90 ± 10.33^d^ (57.29)
8 h	84.00 ± 1.10^a^	447.80 ± 4.50^b^	132.30 ± 6.23^c^ (67.48)	119.40 ± 12.87^c^ (69.81)	209.60 ± 8.02^d^ (43.31)

Values represent mean ± SEM (n = 6).

*Values for a given group in a row followed by same letter as superscript are not significantly different according to Tukey's multiple comparison procedure (at P < 0.05).

Values given in parenthesis are percent blood glucose reduction from the 0 h value in the respective column

3. Toxicity Evaluation

In the acute toxicity study, the methanolic extract of *T. procumbens *did not show any mortality and none of the treated animal showed any visible symptoms of toxicity up to a dose of 5 g/kg body weight. Even at this high dose there was no gross behavioral changes indicating high margins of safty.

### Sub-chronic dose study

1. Effect on Body weight

The results presented in Table 3; exhibit the changes of body weight among diabetic and non-diabetic rats. A normal body weight gain was observed in Group I, II and III. However, a significant decrease of body weight gain was observed at 0 d among the rats dosed with alloxan (Group IV, V, VI and VII). Thereafter, the treated diabetic animals showed sign of recovery in body weight gain. On the contrary, untreated diabetic rats showed a progressive fall in body weight throughout the experimental period. Body weight in both the extract treated daibetic groups was significantly increased from 15 d after dosing. In glibenclamide (10 mg/kg) dosing group, no significant changes compared to that of diabetic control were detected.

**Table 3:** Effects of treatment with *Tridax procumbens *extract on body weight in normal and alloxan diabetic rats.

Group (n = 6)	Dose (mg/kg b.wt)	BODY WEIGHT IN GRAMS					
		
		Alloxan^α) ^dosing	DAYS AFTER DOSING				
			
			0 d^β)^	7 d	15 d	21 d	30 d^γ)^
Normal Control	-	162.6 ± 4.2^a^	201.3 ± 1.9 ^b ^(23.80)	208.4 ± 2.1^b ^(3.52)	217.4 ± 6.2^b ^(4.31)	225.8 ± 4.9^c ^(3.86)	234.4 ± 7.8 ^c ^(3.80)
Normal+ Extract	250	160.0 ± 3.6^a^	196.2 ± 2.7 ^b ^(22.62)	205.5 ± 1.9 ^b ^(4.74)	213.2 ± 5.4 ^c ^(3.74)	221.1 ± 3.2 ^c ^(3.70)	228.6 ± 4.2 ^c ^(3.39)
Normal+ Extract	500	156.1 ± 2.4^a^	197.6 ± 2.1 ^b ^(26.58)	205.1 ± 3.0 ^b ^(3.79)	210.4 ± 4.3 ^b ^(2.58)	218.3 ± 3.7^b ^(3.75)	227.3 ± 2.9^c ^(4.12)
Diabetic Control	-	156.0 ± 2.9^a^	163.4 ± 2.8^b ^(4.74)	160.4 ± 3.2^b ^-(1.83)	155.4 ± 4.0 ^c ^-(3.11)	154.6 ± 5.2^b ^-(0.51)	145.6 ± 5.2^c ^-(5.82)
Diabetic+ Extract	250	157.0 ± 3.1^a^	164.2 ± 1.7^b ^(4.58)	165.4 ± 1.9^b ^(0.73)	167.7 ± 2.8^b ^(1.39)	176.3 ± 3.2^b ^(5.12)	182.6 ± 5.0^c ^(3.57)
Diabetic+ Extract	500	162.9 ± 2.2^a^	171.2 ± 1.4^b ^(5.09)	173.3 ± 2.1^b ^(1.22)	176.5 ± 3.1^b ^(1.84)	183.1 ± 2.5^b ^(3.73)	189.2 ± 6.4^c ^(3.33)
Diabetic+ Glibenc-lamide	10	157.3 ± 1.9^a^	165.3 ± 2.4^b ^(5.08)	167.3 ± 3.0^b ^(1.20)	168.2 ± 2.8^b ^(0.53)	174.0 ± 8.7^b ^(2.83)	176.7 ± 3.8^b ^(1.55)

Values represent mean ± SEM (n = 6).

*Values for a given group in a row followed by same letter as superscript are not significantly different according to Tukey's multiple comparison procedure (at P < 0.05).

^α) ^At alloxan dosing; ^β) ^at initial dosing after fasting (15 d after alloxan dosing); ^γ) ^at sacrifice after fasting

Values given in parenthesis are percent (%) body weight change compared to previous observation of their respective group

2. Effect on Fasting Blood Glucose (FBG) Levels

The Table 4 demonstrate the levels of FBG in normal and alloxan diabetic rats at the time of alloxan dosing, 0 d (just before the drug administration) and 7, 15 and 30 days of treatment. Both the doses of 50% methanolic extract of *T. procumbens *did not cause any significant change in fasting blood glucose levels in normal rats. The administration of *T. procumbens *extract to diabetic rats resulted in a significant decrease in the levels of fasting blood glucose. In *T. procumbens *treated rats, although a significant antihyperglycemic effect was evident from the 7 d onwards, the decrease in FBG was highly pronounced on 30 d and moved towards resettlement to the normal level. The antihyperglycemic effects of *T. procumbens *were more prominent than glibenclamide. Administration of the extract at 500 mg/kg b.wt. dose did not show significant variations in FBG when compared to that of 250 mg/kg b.wt. dose level.

**Table 4:** Effects of treatment with *Tridax procumbens *extract on Fasting blood glucose (FBG) in normal and alloxan diabetic rats.

Group (n = 6)	Dose (mg/kg)	Alloxa^α) ^dosing	Days after dosing				
			0 d^β)^	7 d	15 d	21 d	30 d^γ)^
Normal Control	-	81.21 ± 2.1^a^	79.00 ± 1.8^a^	86.12 ± 2.0^a^	77.45 ± 2.6^a^	83.20 ± 3.1^a^	80.16 ± 3.6^a^
Normal+ Extract	250	78.54 ± 2.4^a^	77.20 ± 2.6^a^	79.20 ± 3.5^a^	76.56 ± 4.8^a^	75.00 ± 3.5^a^	76.60 ± 3.0^a^
Normal+ Extract	500	83.20 ± 1.8^a^	80.63 ± 3.0^a^	84.25 ± 2.9^a^	79.36 ± 6.2^a^	74.25 ± 5.2^a^	73.60 ± 5.8^b^
Diabetic Control	-	80.16 ± 1.6^a^	452.35 ± 4.6^b^	470.20 ± 6.2^b^	459.48 ± 11.2^b^	495.00 ± 9.2^c^	512.40 ± 9.6^c^
Diabetic+ Extract	250	82.00 ± 2.8^a^	454.16 ± 5.4^b^	299.30 ± 10.2^c^ (34.09)	215.00 ± 9.6^d^ (28.16)	186.82 ± 14.7^e^ (13.10)	171.24 ± 12.5^f^ (8.33)
Diabetic+ Extract	500	84.40 ± 2.4^a^	468.20 ± 4.6^b^	289.50 ± 8.6^c^ (38.16)	204.20 ± 12.3^d^ (29.46)	179.42 ± 11.6^e^ (12.13)	166.00 ± 10.2^f^ (7.47)
Diabetic+ Glibenc-lamide	10	79.78 ± 2.1^a^	483.83 ± 6.8^b^	394.14 ± 9.2^c^ (18.53)	293.20 ± 14.3^d^ (25.61)	258.40 ± 12.9^e^ (11.86)	242.00 ± 11.4^e^ (6.34)

Values represent mean ± SEM (n = 6).

*Values for a given group in a row followed by same letter as superscript are not significantly different according to Tukey's multiple comparison procedure (at P < 0.05).

^α) ^At alloxan dosing; ^β) ^at initial dosing after fasting (15 d after alloxan dosing); ^γ) ^at sacrifice after fasting

Values given in parenthesis are percent (%) glucose reduction, compared to previous observation of their respective group (in rows)

3. Effect on Oral Glucose Tolerance Test (OGTT)

Table 5 shows the changes in the levels of blood glucose in normal and diabetic groups after oral administration of glucose (3 g/kg). The data of OGTT revealed that the blood glucose levels of the normal rats reached peak at 60 min after the oral glucose load and gradually decreased to the pre-glucose load level. A better glucose tolerance pattern was observed in normoglycemic extract treated animals, when compared the normal control rats. In diabetic control group, highly impaired glucose tolerance was evident. However, in the extract treated diabetic rats, significant blood glucose attenuation was observed from 60 min onwards.

**Table 5:** Effects of treatment with Tridax procumbens extract on oral glucose tolerance (OGTT) after 30 days of drug administration in normal and alloxan diabetic rats.

Group (n = 6)	Dose (mg/kg b wt)	Blood Glucose levels (mg/dl)					
		-30 min	0 min	30 min	60 min	90 min	120 min
Normal Control	-	79.12 ± 2.8^a^	80.16 ± 3.6^a^	146.5 ± 9.6^b^	152.4 ± 8.4^b^	121.6 ± 21.6^c^	102.3 ± 32.0^d^
Normal+ Extract	250	78.54 ± 4.1^a^	76.60 ± 3.0^a^	151.0 ± 11.7^b^	143.6 ± 9.6^b^	119.2 ± 16.3^c^	87.5 ± 12.3^d^
Normal+ Extract	500	79.58 ± 3.2^a^	73.60 ± 5.8^a^	149.4 ± 8.2^b^	136.6 ± 11.0^c^	120.6 ± 11.6^c^	84.6 ± 19.0^d^
Diabetic Control	-	508.2 ± 4.7^a^	512.40 ± 9.6^a^	HI^b^	597.2 ± 3.4^b^	580.6 ± 32.0^b^	547.5 ± 9.2^c^
Diabetic+ Extract	250	186.6 ± 2.4^a^	181.24 ± 12.5^a^	299.8 ± 43.0^b^	261.0 ± 34.2^c^	196.4 ± 14.9^d^	169.6 ± 14.4^e^
Diabetic+ Extract	500	163.0 ± 6.8^a^	165.00 ± 10.2^a^	302.4 ± 19.6^b^	271.0 ± 18.3^c^	190.4 ± 28.4^d^	160.3 ± 18.4^e^
Diabetic+ Glibenc-lamide	10	190.4 ± 4.6^a^	190.00 ± 11.4^a^	285.6 ± 46.0^b^	256.6 ± 29.4^c^	231.0 ± 17.2^c^	199.6 ± 10.3^d^

Values represent mean ± SEM (n = 6).

*Values for a given group in a row followed by same letter as superscript are not significantly different according to Tukey's multiple comparison procedure (at P < 0.05).

HI = High Blood Glucose Level i. e. above 600 mg/dl

## Discussion

Alloxan, a beta cytotoxin induces diabetes by free radical generation, which causes a massive reduction of the insulin secreting β-cells of the islets of langerhans, resulting in a decrease in endogenous insulin release, which paves the ways for the decreased utilization of glucose by the tissue [20]. Glibenclamide is an oral sulphonylurea anti-diabetic preparation and widely used as reference drug in anti-diabetic activity test [21,22].

The data obtained clearly indicate that the acute and sub-chronic oral administration of *T. procumbens *extract exhibited only anti hyperglycaemic effects and did not produce any change in the blood glucose levels of normoglycemic fasted rats. Further, it was also found that the sub-chronic treatment with *T. procumbens *extract for a period of 30 days produced a significant decrease in fasting blood glucose level and improved glucose tolerance of alloxan diabetic rats as well as in glucose-loaded normal rats. The drug showed optimum activity at 250 mg/kg and further increase in extract dose did not result in a further significant decline in blood glucose levels, thus it appears that unlike insulin and other common hypoglycaemic agents overdose of the drug may not result in hypoglycemia.

In the acute study, the anti-hyperglycemic action of the *T. procumbens *was better (higher in percent and close to normal range) than that of the standard drug glibenclamide. The possible mechanism by which *T. procumbens *brings about its anti-diabetic action may be by potentiating the insulin effect of plasma by stimulating insulin release from the remnant pancreatic β-cells or its release from the bound form [23]. Beside this, it might involve extra-pancreatic action in these alloxan-diabetic rats, which might include the stimulation of peripheral glucose utilization or enhancing glycolytic and glycogenic processes with concomitant decrease in glycogenolysis and gluconeogenesis [24]. In the above context a number of other plants have also shown similar hypoglycemic mechanism [6,25-28].

In diabetic rats, there was a decline in body weight compared to normal rats. Normal body weight gain is indicator of efficient glucose homeostasis; but in diabetics, glucose is not available therefore the cells use alternatively proteins for energy, consequently due to excessive breakdown of tissue protein (muscle wasting) a loss in body weight occurs. Treatment with *T. procumbens *induced an increase in the body weight in diabetic rats, which can be attributed to the improvement in insulin secretion and glycemic control. Similar effect on body weight gain was previously reported with other plants, well known for their anti-diabetic activity[29].

The antihyperglycemic activity of *T. procumbens *might be due to individual or synergistic activity of flavonoids and other active phytoconstituents of the plant.

On the basis of the current investigation it was noted that the methanolic extract of *T. procumbens *acted in a similar fashion to glibenclamide (standard drug) and it can be suggested that these results provide pharmacological evidence for its folklore claim as an anti-diabetic agent.

## Conclusion

From the present study, it is concluded that *Tridax procumbens *may be useful in treating diabetes mellitus with no visible signs or symptoms of toxicity in normal rats indicating a high margin of safety. The 50% methanolic extracts of *Tridax procumbens *have indicated high level of anti-diabetic activity. The extracts exhibited anti-hyperglycemic activity comparable to that of a standard anti-diabetic drug, glibenclamide. The traditional use of *Tridax procumbens *to treat diabetes is supported by laboratory results from this study, suggesting a need to isolate and evaluate active constituents responsible for the exhibited biological activity.

## Competing interests

The authors declare that they have no competing interests.

## Authors' contributions

HP conceived of the study, and participated in its design and coordination and drafted the manuscript. SS carried out the experiment and performed the statistical analysis. BSK collected the plant material and prepared the extract, participated in the biochemical estimations. KJ participated in the biochemical estimations. GCJ participated in the design of the study and helped to draft the manuscript. All authors read and approved the final manuscript.

## Pre-publication history

The pre-publication history for this paper can be accessed here:

http://www.biomedcentral.com/1472-6882/9/48/prepub
